# Supplementary figures and images for: Analysis of Tick Surface Decontamination Methods
Source: Microorganisms. 2020 Jun 30;8(7):987. doi: 10.3390/microorganisms8070987 (PMC7409031; doi:10.3390/microorganisms8070987)

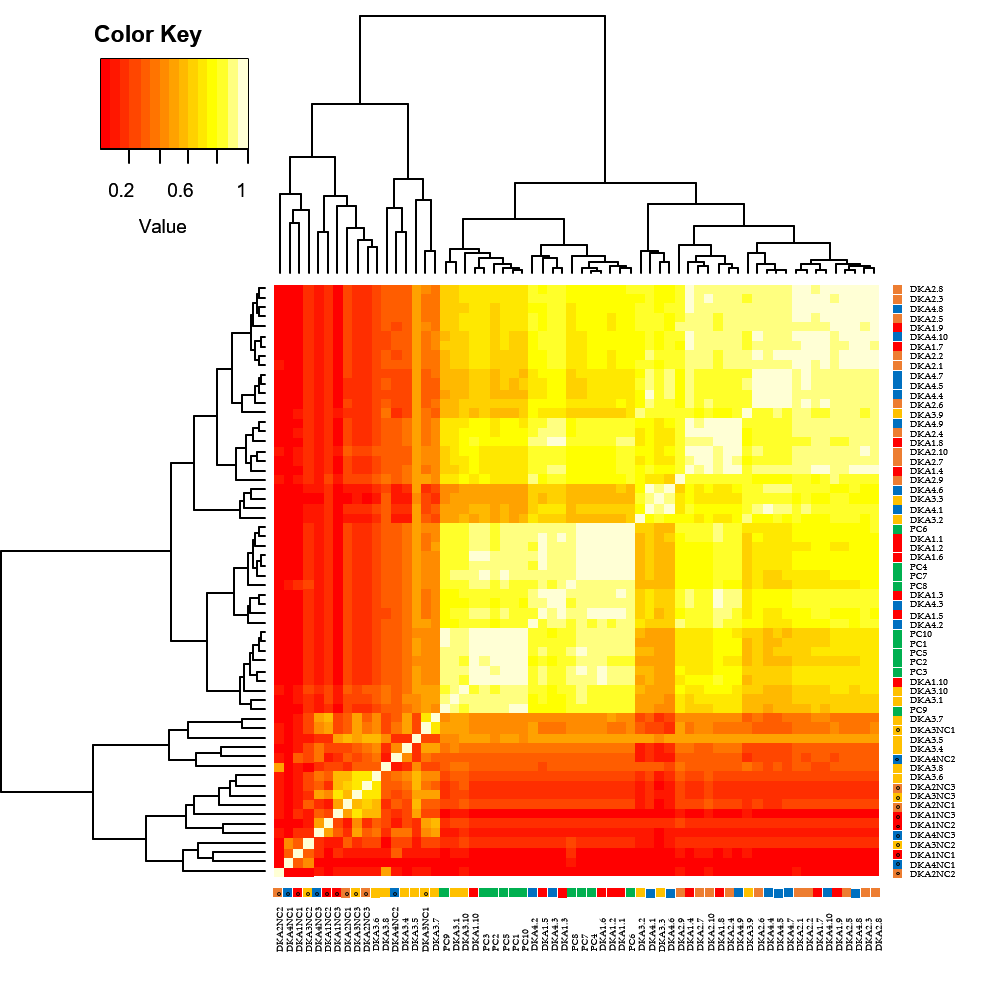

Supplement: Supplementary file 1 [file microorganisms-08-00987-s001.zip › Supplementary figures/Supplementary Figure S1A_BrayCurtisSimilarity_DNA.tif]

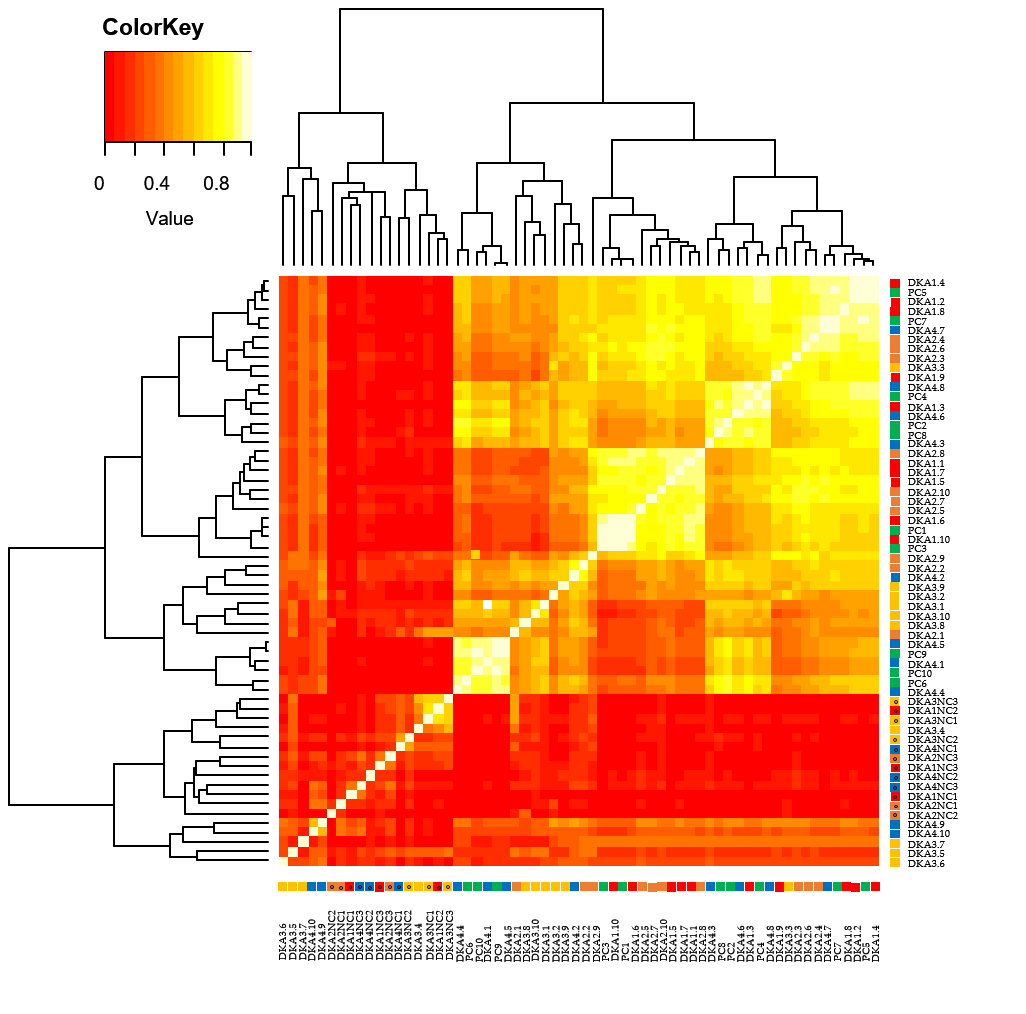

Supplement: Supplementary file 1 [file microorganisms-08-00987-s001.zip › Supplementary figures/Supplementary Figure S1B_BrayCurtisSimilarity_cDNA.tif]

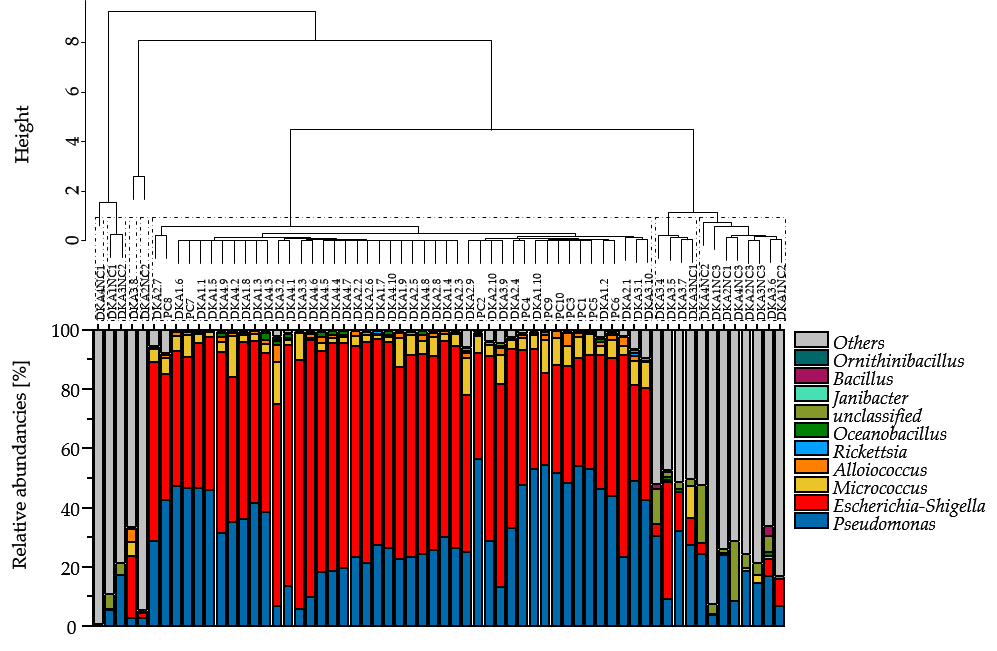

Supplement: Supplementary file 1 [file microorganisms-08-00987-s001.zip › Supplementary figures/Supplementary Figure S2A_EuclideanDistances_relAbu_single_DNA.tif]

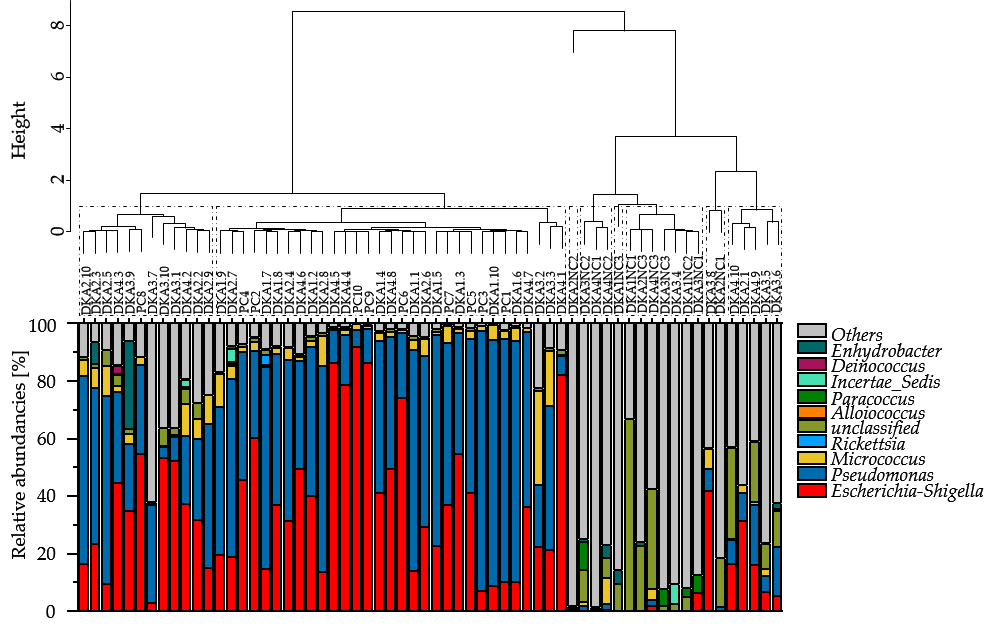

Supplement: Supplementary file 1 [file microorganisms-08-00987-s001.zip › Supplementary figures/Supplementary Figure S2B_EuclideanDistances_relAbu_single_cDNA.tif]
